# Therapeutic strategies for BRAF mutation in non-small cell lung cancer: a review

**DOI:** 10.3389/fonc.2023.1141876

**Published:** 2023-08-14

**Authors:** Megha Puri, Kunal Gawri, Richa Dawar

**Affiliations:** ^1^Department of Internal Medicine, Saint Peter’s University Hospital, New Brunswick, NJ, United States; ^2^Department of Pulmonary, Critical Care and Sleep Medicine, University of Buffalo, Buffalo, NY, United States; ^3^Sylvester Comprehensive Cancer Center, University of Miami Health System, Miami, FL, United States

**Keywords:** non-small cell lung cancer, BRAF mutation, BRAF mutation V600, non-V600 mutation, lung cancer

## Abstract

Lung cancer is the leading cause of cancer related deaths. Among the two broad types of lung cancer, non-small cell lung cancer accounts for 85% of the cases. The study of the genetic alteration has facilitated the development of targeted therapeutic interventions. Some of the molecular alterations which are important targets for drug therapy include Kirsten rat sarcoma (KRAS), Epidermal Growth Factor Receptor (EGFR), V-RAF murine sarcoma viral oncogene homolog B (BRAF), anaplastic lymphoma kinase gene (ALK). In the setting of extensive on-going clinical trials, it is imperative to periodically review the advancements and the newer drug therapies being available. Among all mutations, BRAF mutation is common with incidence being 8% overall and 1.5 – 4% in NSCLC. Here, we have summarized the BRAF mutation types and reviewed the various drug therapy available - for both V600 and nonV600 group; the mechanism of resistance to BRAF inhibitors and strategies to overcome it; the significance of comprehensive profiling of concurrent mutations, and the role of immune checkpoint inhibitor in BRAF mutated NSCLC. We have also included the currently ongoing clinical trials and recent advancements including combination therapy that would play a role in improving the overall survival and outcome of NSCLC.

## Introduction

Lung cancer is the second most common cancer worldwide. It is the leading cause of cancer-related deaths accounting for 18% of all cancer related deaths (1, 2). In United states alone, it will account for estimated 127,070 deaths in the year 2023 (3). Among the two major subtypes, Non-small cell lung cancer (NSCLC) accounts for 85% of the cases (4). NSCLC is significantly more common than small cell lung cancer (SCLC) and is further subdivided into squamous and non-squamous histological types (5). Although the classification between small cell and non-small cell is still widely used, molecular classification demonstrates that this histological classification is no longer appropriate to guide therapy. NSCLC comprises a group of heterogenous tumors having varied genetic alterations.

The most common genetic alteration associated with NSCLC is Kirsten rat sarcoma (KRAS) and Epidermal Growth Factor Receptor (EGFR) gene. They are involved in tumor initiation and are important targets for drug therapy. Other important mutations observed are anaplastic lymphoma kinase gene (ALK) rearrangement, C-ROS oncogene 1 (ROS1). Certain molecular alterations identified involve hepatocyte growth factor receptor (MET) and human epidermal growth factor receptor 2 (HER2) genes, rearranged during transfection (RET) gene, V-RAF murine sarcoma viral oncogene homolog B (BRAF) and neurotrophic tropomyosin receptor kinase (NTRK) gene (6). Mutations in tumor protein 53 (TP53) have been observed in advanced stages of NSCLC and is poor prognostic marker (7). The understanding of the pathogenic alterations has led to advancement in the therapeutic intervention available especially combination drug therapy and immune checkpoint inhibitors (1, 8).

## BRAF mutation

BRAF belongs to rapidly accelerated fibrosarcoma (RAF) group of serine threonine group of kinases and is significantly involved in cell proliferation and differentiation through the mitogen activated protein kinase (MAPK) signaling pathway. The rat sarcoma (RAS)/RAF-MAPK extracellular signal regulated kinase (ERK)-MAPK pathway can get activated by mutation at various levels in the pathway. The various levels of mutation have been seen in multiple cancers including melanoma, NSCLC, papillary thyroid cancer and colorectal cancer and around 300 distinct BRAF mutations have been identified (9–11). The incidence of BRAF mutation in all human cancers is around 8% with the incidence in NSCLC being 1.5 - 4% (12–15).

**Figure 1:**
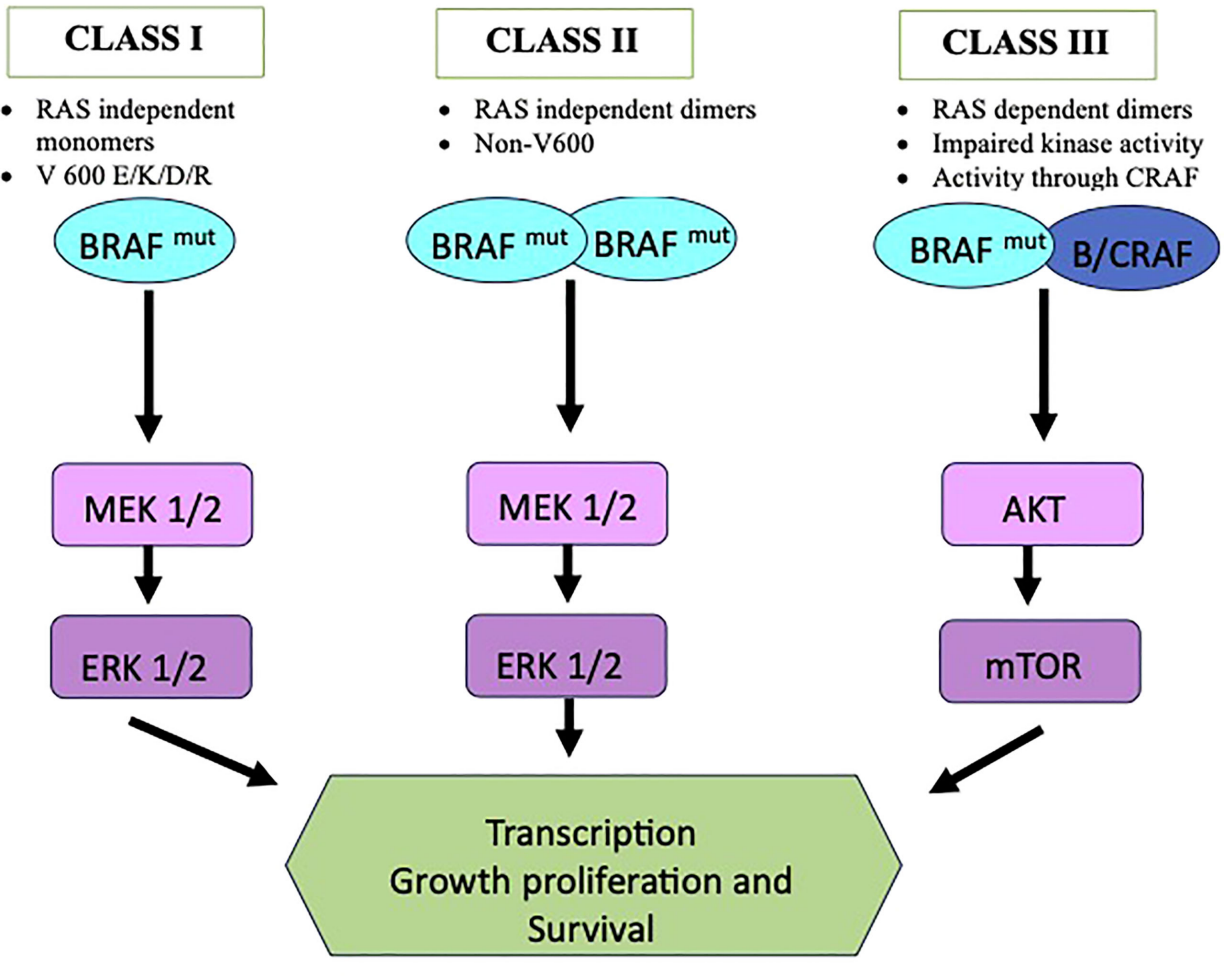
Classification of BRAF mutations.

The mutations are broadly named as V600 codon and non-V600 codon mutations and are divided into 3 categories as shown in **Figure 1**. Class I mutant are constitutive active RAS independent monomers that involves codon 600 (including V600 E/K/D/R) causing strong activation of BRAF kinase. Class II comprises constitutive active RAS independent dimers outside codon 600 (including K601, L597, G464, and G469 mutations) and are located in the P loop segment. Class III includes RAS dependent dimers and have impaired kinase activity. In this case the activity of MAPK pathway is enhanced *via* raf-1 protooncogene CRAF activation (16, 17). Class II and III are more prevalent for certain tumor types (18). In clinical practice BRAF mutations are commonly classified as V600 and non-V600 mutations. V600 have been seen more in female gender and has been seen as a negative prognostic factor and non-V600 mutations are more seen in male gender (19, 20). BRAF mutations, especially nonV600 have been associated with history of smoking and have more propensity towards central nervous system (CNS) involvement. Class I mutations, however, have shown lower incidence of brain metastasis at the time of diagnosis. Among the V600 codon mutations, around 20-30% have no smoking history. V600 mutations is also associated with shorter disease-free survival period (21, 22). The differences observed between the V600 mutation, mainly V600E, and Non-V600 have been summarized in **Table 1**.

**Table 1:** Difference between V600E mutation and non-V600E mutations.

V600E mutations	Non- V600E mutations
More seen in Female gender	Almost exclusively in male gender
More likely in never smokers. Around 20-30% had no smoking history.	Associated with history of smoking.
Shorter disease-free survival period and overall survival rate	Relatively longer disease-free survival period.
Negative prognostic factor	Relatively positive prognostic factor
Relatively less propensity towards Central Nervous system (CNS) involvement	More propensity towards CNS involvement
More aggressive histologic types seen, like micropapillary.	Higher mutational burden, hence better response to immunotherapy

## BRAF target drug therapy

BRAF inhibitors available are namely Sorafenib, Vemurafenib, Dabrafenib and Encarafenib. MEK inhibitors namely, Trametinib, Cobimetinib, Bimetinib block the ERK signaling in the MAPK pathway and delays the emergence of resistance due to MAPK pathway reactivation (23).

Sofrafenib has weak activity for mutant BRAF and also has significant toxic effects. Vemurafenib (PLX4032) is a inhibitor of mutated V600-BRAF (24). The side effects are dose related and most common observed are rash, arthralgia, nausea, fatigue, photosensitivity, pruritus, palmar-plantar dysesthesia and cutaneous squamous cell carcinoma. A dose of 960 mg twice daily has been determined to be tolerable (25). Dabrafenib is ATP-competitive BRAF kinase inhibitor. The most common adverse effects observed are fatigue, pyrexia and cutaneous squamous cell carcinoma (26). Trametinib (GSK1120212) is a non-ATP competitive inhibitor of both MEK1 and MEK2 (27). The common toxicity observed are diarrhea, peripheral edema, skin related toxicity. The cardiac and hepatic events observed have been reported to be reversible on discontinuation of trametinib (28).

Very few studies are available in the context of Sofrafenib (29). Carter et al (30) used Sorafenib in patients who had received chemotherapy. The idea behind it was the combination could delay tumor growth without increasing toxicity. Certain other trials have been done but did not test for BRAF mutation status (31, 32). It remains largely unexplored.

In the VE Basket trial, Vemurafenib 960 mg twice a day was given to 62 patients with NSCLC having V600 mutations. It was a phase II study. Out of the 62 patients, 54 were pretreated and 8 were naïve. It was found that objective response rate (ORR) was similar in both the groups – 37.5 in naïve and 37% in pretreated group. The median progression-free survival (mPFS) was 6.1 and 12.9 respectively in pretreated and naïve groups whereas median overall survival (mOS) was 15.4 in the pretreated group and not reached in the naïve group (33).

The EURAF study done by Gautschi et al. comprised of 35 patients. Out of these, 29 harbored V600 mutation and 6 were nonV600. 5 patients were given BRAF inhibitor (BRAF-i) as first line and 30 were given as subsequent line. So total of 39 lines of BRAF-i were counted in the trial. 29 of the patients received Vemurafenib, 9 received Dabrafenib and 1 received Sofrafenib. The ORR in the V600 group treated with Vemurafenib (n=24) was 54% and disease control rate (DCR) 96%. Overall, the study showed mPFS of 5 months and OS of 10.8 months. In the nonV600 group, only a partial response and poor outcome was seen (34).

Another research by Mazieres et al. used Vemurafenib and all the patients had more than one line of treatment. In the V600 mutation group the mean ORR was 44.9, mPFS 5.2 months and mOS 10 months. In the nonV600 mutation group, no tumor response was seen and the mPFS was 1.8 months (35).

Planchard et al. conducted a phase II non-randomized controlled trial with Dabrafenib in advanced V600 positive NSCLC. They conducted the trial under 3 separated arms. Group A has 84 patients and used Dabrafenib monotherapy. Out of these, 6 were T/t naïve and 78 had received prior systemic therapy. The ORR and DCR for pretreated group was 33% and 58% respectively. Of the T/t naïve, 4 out of 6 had treatment response. Those treated with 1-3 previous treatment lines had mPFS of 5.5 months and mOS of 12.7 months. The Group B used combination Dabrafenib and Trametinib in 57 pretreated V600 positive patients. The median follow up was 16.6 months. The ORR was 68%, mPFS 10.2 months, OS 18.2 months, DCR 81% and duration of response (DoR) 9.8 months. Five-year survival rate was 19%. The Group C used the combination Dabrafenib-Trametinib therapy in 36 treatment naïve patients. The median follow up was 16.3 months. The ORR was found to be 64%, mFS 10.8 months, OS 17.3 months, DoR 10.2 months and the 5-year survival rate 22% (36–39).

Current guidelines recommend the combination of Dabrafenib with Trametinib for BRAF V600 positive NSCLC (23). The studies have been summarized in **Table 2**.

**Table 2:** Studies in targeted therapy for BRAF-mutant NSCLC.

Name	Study Design	BRAF-I used	Number of patients	Lines of treatment	mPFS (months)	mOS or OS (months)	ORR (%)
VE Basket Trial	Phase 2	Vemurafenib	62	54 pretreated	6.1	15.4	37
				8 naive	12.9	Did not reach	37.5
EURAF	Retrospective	Vemurafenib	24	Pretreated + naive	5	10.8	54%
		Dabrafenib	9				
		Sofrafenib	1				
Mazieres et al	Phase 2	Vemurafenib	101 V600, 17 non-V600	Pretreated	5.2 1.8	10 5.2	44.9 0 (cohort stopped)
Planchard et al Cohort A:	Phase 2	Dabrafenib	84	78 pretreated 6 naive	5.5 NA	12.7 NA	33 NA
Cohort B:		Dabrafenib + Trametinib	57	Pretreated	10.2	18.2	68
Cohort C:		Dabrafenib + Trametinib	36	Naive	10.8	17.3	64

NA, Not applicable.

## Resistance to BRAF kinase inhibitors

Despite the advances in the available BRAF inhibitors, the disease progression will eventually occur with development of either *de novo* or acquired BRAF pathway inhibitor resistance. Delineation of resistance mechanism to elucidate alternative drug targets could assist in formulation alternate drug strategies. The bypass activation mutation seems to be the main cause of resistance, out of which the most common are found in central MAPK nodes and lead to MAPK reactivation (40). CRAF and A- raf (ARAF) protooncogene isoforms have been observed in melanoma, which could cause MAPK pathway activation once BRAF is inhibited and cause BRAF-i resistance (41). The COT/TPL2 (MAP3K8) expression has also been observed to cause *de novo* resistance in melanoma cell lines (42). In BRAF V600 NSCLC, the activating mutations like KRAS or neuroblastoma RAS oncogene (NRAS) after use of BRAF-i or dual blockade with dabrafenib and trametinib often have been observed as causing resistance. These mutation bypasses the BRAF-V600 inhibition and leads to activation of downstream MAPK pathway (43–45).

Resistance mechanism could also involve MAPK pathway through reactivation of ERK signaling through BRAF splice variants or BRAF gene amplification (12, 46). An institutional prospective trial MATCH-R (“Matching Resistance”) revealed potential mutation responsible for resistance, namely, MEK1, NRAS Q61K, KRAS Q61R, and K57N (47). Sheikine et al. identified new post-treatment mutations, that could corelated to acquired resistance. They reported mutations involving KRAS (G12R, K61H, G12D, V141), NRAS (Q61K), a rearrangement in the setting of V600E, biallelic inactivation of SMARCA4 and a homozygous deletion of MAPK2K4 (48). Some less common mechanism could involve other pathways, like activation of PI3K/mTOR through mutations in AKT activation and loss of function of PTEN (12).

The expression of a ligand dependent stimulation of RTKs and p61(aberrant BRAF V600 splice form) are other potential mechanism of MAPK pathway reactivation. Activation of signaling pathway, like phosphatidylinositol-3-kinase – protein kinase B – mammalian target of rapamycin (PI3K/AKT/mTOR) have also been described (49, 50). A case with protein kinase B (AKT) mutation was reported in BRAF-V600 mutant NSCLC. The resistance occurs through engagement of EGFR signaling. The resistance might be overcome by using combined BRAF-i and EGFR inhibitors (49). Autocrine activation of fibroblast growth factor receptor (FGFR) leads to sustained extracellular signal-regulated kinases (ERK) activation and has been more commonly seen in dual BRAF/MEK inhibitor resistance. The potential utility of FGFR inhibitors in such cases could be a promising strategy (50). Planchard et al. had reported presence of co-mutations and alteration of phosphatidylinositol-3-kinase (PI3K) pathway as a negative prognostic factor (39).

**Figure 2:**
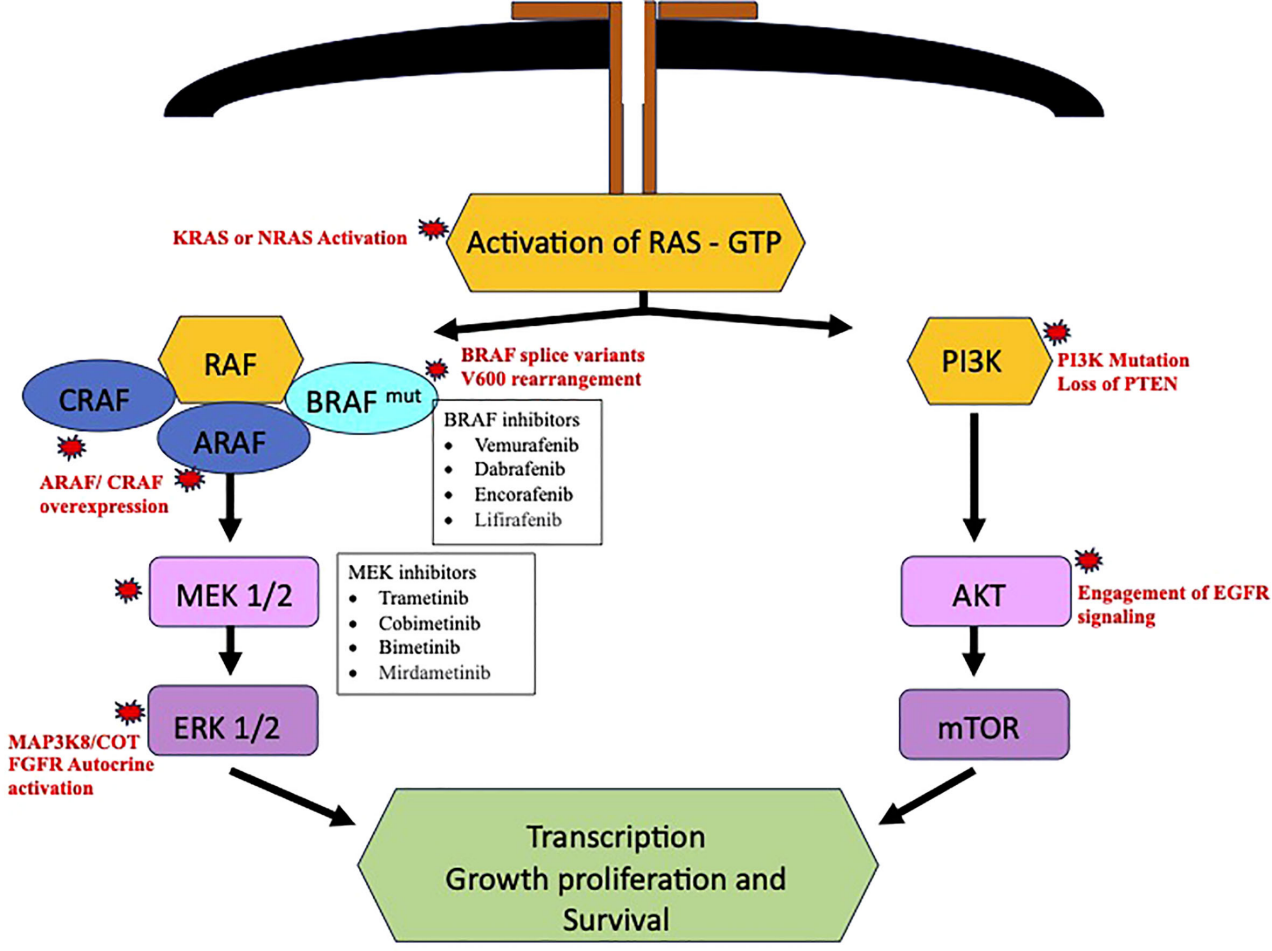
RAF activation pathway and potential Resistance Mechanism.

Rudin et al. reported a patient to have developed KRAS (G12D) mutation after use of Dabrafenib. Secondary mutations in TP53 and cyclin dependent kinase inhibitor 2A (CDKN2A) were also found. They are not directly related to RAF-dependent pathway and role to the resistance attained is not clear (43). A case of metastatic lung adenocarcinoma was reported by Abravanel et al. wherein the patient received a combination therapy with dabrafenib and trametinib. Initially significant response to therapy was seen, however, within 21 weeks of therapy the patient’s disease progressed and was found to have acquired NRAS-Q61K mutation (44). This patient also has remote history of breast cancer, could the presence/history of another malignancy influence the acquisition of the resistance mutations could be further studied. The RAF activation pathway with potential resistance mechanisms is summarized in **Figure 2**.

It is imperative to do a comprehensive profiling of the possible co-occurring mutations, to detect the presence of acquired resistance. It would play a significant role in choosing the currently available targeted therapies and guide the scientific community in designing clinical trials targeting the specific resistance. A multi-gene testing panel rather than a single gene panel is more suitable to detect the mutations. Some of the methods to detect this resistance mechanism include analyses of circulating tumor DNA (ctDNA) (51). Liquid biopsy is a non-invasive analysis using either ctDNA or plasma circulating tumor cells. Analytes could also include circulating cell-free DNA or RNA, tumor educated platelets and circulating extracellular vesicles. This method resolves the issues of tissue scarcity and damage associated with the standard tissue biopsy (52, 53). Currently, real time polymerase chain reaction (PCR) and Next generation sequencing (NGS) are the most used methods. It can facilitate testing of multiple biomarkers with use of small amount of tumor tissue. However, the use of NGS has its own drawbacks. It has a high turnaround time and requires use of expensive and high-end equipment and reagents; hence it is not yet accessible all round the world. Cohen et al. reported that combination of sequential DNA NGS and RNA NGS is the most efficient strategy for mutation detection in smoking associated NSCLC and recommended a parallel approach for never smokers (54). In current practice, all metastatic or locally advanced NSCLC should be tested for driver mutations including BRAF, MET, RET, NTRK, KRAS, HER2 if common mutations EGFR, ALK and ROS protooncogene are negative (55, 56).

## Comprehensive profiling of concurrent mutations

The rate of presence of concurrent mutations in BRAF mutated NSCLC has been reported to have a wide range from 14.3 to 30.2%. It is much higher than concurrent mutation rate seen in other driver mutations (5%) (57–59). Qu et al. reported TP53 to be the most common occurring co-mutation, 6 out of 53 (11.3%) patients included in the study (59). Mayall et al. also reported TP53 to be the most common concurrent mutation (5 out of 8 patients) in the cohort studied. There was no similarity observed in any of the observed five mutations, each of the mutation was unique. The alterations noted were C343F (725 G>T), R248L (743 G>T), E298X (892 G>T), I195T, and splice site 559 + 1 G>C (60). In a retrospective analysis by Krohn et al. in 174 patients, co-occurring mutations were found in 70% (121) BRAF mutated patients. TP53 was found as the most frequent (74%, 89 patients) co-alteration (61). The co-occurrence of other mutations seen with BRAF have been EGFR, PI3KA, KRAS, ALK translocation, c-MET amplification, MSH2 mutation, AXIN2 mutation (15, 19, 59). BRAF V600 and non-V600 mutated NSCLC, both have been reported to have concurrent mutations. Cardarella et al (15) reported presence of KRAS in nonV600 NSCLC and Kinno et al (62) reported presence of EGFR along with non-V600 BRAF mutated NSCLC. It would be interesting to explore whether BRAF are the primary or secondary oncogenic driver mutations in these cases.

Patients with double mutations have been found to have inferior overall survival compared with single BRAF mutation. TP53 and PI3KA co-mutation carries a negative prognosis (39, 58, 59). Hence, important is the role of PCR or NGS in multiplex genotyping for comprehensive profiling of the NSCLC patients to decipher the presence of concurrent mutations. To target co-mutations associated with BRAF, double or triple targeted therapy is used. EGFR targeting tyrosine kinase inhibitors (TKI) are used NSCLC having BRAF plus EGFR mutations. KRAS co-mutation was given Dabrafenib-Trametinib therapy (59). The use of immunotherapy, specifically immune checkpoint inhibitors (ICIs), is more and more being used as a solution to BRAF co-mutations (63). More research is needed to study the clinical implications of the use of multi-targeted chemotherapy drugs and immune therapy in concurrent mutations.

## Immune checkpoint inhibitor therapy - monotherapy vs combination therapy

Very limited studies in the form of retrospective studies demonstrated Programmed cell death ligand 1 (PD-L1) positivity in NSCLC with BRAF mutations (64–66). Dudnik et al. demonstrated that use immune checkpoint inhibitors (ICIs) in BRAF mutated patients had a limited response and is similar to the unselected population. They included 39 BRAF mutated patients treated with ICIs. Out which 22 (12 V600 and 10 nonV600) - 10 with Pembrolizumab, 11 with Nivolumab and 1 with atezolizumab. The ORR 25% and 33% and mPFS was 3.7% and 4.1% respectively for the two groups. mOS was not achieved in either of the groups. A low/intermediate tumor mutation burden (TMB) and microsatellite-stable status was found in BRAF mutated NSCLC patients (67). Several other studies also observed a similar response. One study showed ORR, mPFS and mOS of 28.1%, 3.0 and 13.1 months, respectively treated with ICIs in second line of treatment (68). The ORR of PDL1 inhibitor as the only therapy in BRAF-mutant patients is about 10%–30%, with a mPFS of 2–4 months. Guisier et al. supported that the PDL1 inhibitor being used as a second-line ICI monotherapy is similar in to wild-type NSCLC (69). Similar results were obtained by Rihawi et al (70). Offin et al. demonstrated improved ORR of 22% and OS 2.4 years in non-V600 mutations when compared to V600 mutations (71). The non-V600 mutated NSCLC are seen more in non-smoker and have been found to have increased TMB and hence, a better response to ICIs (71–73). In summary, these data indicated limited efficacy of ICIs in BRAF mutant NSCLC. However, the efficacy of PDL1 inhibitors in patients of advanced NSCLC has been noted. This could be attributed to an increased expression of PDL1 in BRAF mutant NSCLC in comparison to wild type (67, 72).

Some of the ongoing trials are exploring a combination regimen of ICIs and targeted BRAF-I and MEK-inhibitor (MEK-i) therapies. The concept is based on the fact that PDL1 expression has been found in BRAF mutants and BRAF-i and MEK-i improve T cell mediated immune activation. The preclinical data suggested that inhibition of BRAF and MEK pathway leads to increased activity of CD4 and CD8 T cells and thereby ability to destroy tumor cells. It also leads to increased granzyme B and perforin levels along with increased expression of cytotoxic T-lymphocyte-associated protein 4 (CTLA4) (74–77). The use of anti-CTLA-4 and MEK-i like Selumetinib and Trametinib has shown to provide survival benefit in murine K-ras bearing tumors (78, 79). A stage IV V600 BRAF mutant NSCLC was reported to have achieve a longer period of response when combination of Atezolizumab and chemotherapy was used (80). Hellmann et al. investigated the combination cobimetinib and atezolizumab in patients with solid tumors covering colorectal cancer, melanoma and NSCLC. Out of the total 152 participants, 28 were NSCLC patients and were found to have ORR of 18%, mOS of 13.2 months. The 12-month PFS and OS for NSCLC was 29% and 57% respectively (81). The enrollment for an ongoing randomized phase II/II study, the B-FAST trial (NCT03178552), is currently underway for patients with advanced or metastatic NSCLC that are found to harbor somatic mutations of have TMB using blood based NGS ctDNA assay. In the cohort E of this trial, the V600 mutant patients are being given the triplet of Vemurafenib, cobimetinib and atezolizumab after a run-in period of BRAF-i/MEK-i combination. This preliminary data about combination ICI-BRAF-i/MEK-i targeted therapy warrants further studies to establish an appropriate drug combination and safety and clinical efficacy (82). Some of the ICIs have been summarized in **Table 3**.

**Table 3:** Immune checkpoint inhibitors in BRAF-mutant NSCLC.

Trial	Study Design	ICI	Number of patient/lines	mPFS (s)	mOS (months)	ORR (%
Dudnek	Retrospective	10 Pembrolizumab 11 Nivolumab 1 Atezolizumab	12 V600 10 non-V600	3.7 4.1	NA NA	25 33
Mazieres	Retrospective	ICI	43	3.1	13.6	24
Guisier	Retrospective					
Offin	Retrospective		10 V600 36 nonV600	NA NA	NA 28.8	10 22
Hellman	Phase 1/1b	Cobimetinib and Atezolizumab	28 NSCLC	29%	13.2	18

NA, Not applicable.

## Recent advances and future prospectives

On June 22, 2022, the FDA granted accelerated approval to dabrafenib- trametinib combination for the treatment of all patients more than or equal to 6 years of age with unresectable or metastatic BRAF-V600E mutant solid tumors, who have no alternate treatment option and have progressed following prior treatment. It is not indicated for wild-type BRAF solid tumors. It was based on evaluation of 131 adult patients from open-label, cohort trials BRF117019 (NCT02034110) and NCI-MATCH (NCT02465060), 36 pediatric patients from CTMT212X2101 (NCT02124772), and supported by results in COMBI-v, COMBI-d, and BRF113928. From the adult patient group, 54 (41% 95% confidence interval of 33,50) experienced ORR. The most common adverse reactions in the adults were nausea, rash, fever, chills, fatigue, headache, myalgia, constipation, diarrhea, arthralgia and edema. In adults, the dose recommended for dabrafenib is 150 mg oral twice daily with Trametinib 2 mg oral once daily (83).

PHAROS trial (84) is an open-label, non-randomized, Phase 2 at a multicenter level is going on to determine the safety and efficacy of Encorafenib in combination with Binimetinib in NSCLC patients with BRAF V600 mutation. The patients who were treatment naïve, or first line treatment with an anti-PD1 given as monotherapy of with a platinum-based chemotherapy were enrolled. A total of 98 patients have been recruited. The doses used were 450 mg once daily of Encorafenib and 45 mg twice daily of Binimetinib for a 28-day cycle. Primary set endpoint of ORR was met. Currently, the combination is approved for use in BRAF -V600 positive melanoma patients. A supplemental new drug application (SNDA) is currently under review by the FDA for patients with metastatic NSCLC w a BRAF V600 mutation (85, 86).

ENCO-BRAF trial is another Phase 2 trial currently recruiting patients to assess the Encorafenib and Binimetinib on the same dosing schedule (87).

LXH254 (BRAF inhibitor) plus LTT462 (ERK ½ inhibitor) is being explored for its efficacy in advanced metastatic K-ras or BRAF-mutant NSCLC under clinical trial NCT02974725 (88).

Lifirafenib (BGB-283) is a novel RAF kinase and EGFR inhibitor and antitumor activity in B-RAF mutated solid tumors and KRAS mutant NSCLC with tolerable adverse effects. Future exploration is warranted to explore lifiranib monotherapy of combination therapy in patient with BRAF-i resistance and harbouring RAS mutations (89).

Another Phase I multicenter study (NCT03284502) is exploring the dose, safety and pharmacokinetics of HM95573 in combination with either Cobimetinib or Cetuximab in locally advanced or metastatic Solid Tumors (90).

Some of the current ongoing trials have been summarized in **Table 4**.

**Table 4:** Ongoing Clinical trials with targeted therapy.

Clinical Trial	Study Design	Experiemental Drug	Type of BRAF incuded	Patient population	Status
PHAROS (84, 91) (NCT03915951)	Open label, phase 2 multicenter	Encorafenib (BRAF inhibitor) with Binimetinib (MEK inhibitor)	BRAF V600	Treatment naïve or post anti-PD1 treatment.	Active, Not recruting SNDA under FDA review
ENCO-BRAF (87) (NCT04526782)	Open label, phase 2, multicenter multicohort	Encorafenib with Binimetinib	BRAF V600	Treatment naïve or pretreated.	Recruiting
OCEAN II (92) (NCT05195632)	Open label, phase 2, single arm	Encorafenib with Binimetinib	BRAF V600E	BRAF and MEK-inhibitor treatment naïve; First or second line	Recruiting
NCT03905148 (93)	Phase 1b, Open labe	Lifirafenib (RAF inhibitor) and Mirdametinib (MEK inhibitor)	All advanced tumors including BRAF mutant NSCLC	Advanced or metastatic tumor	Recruiting
LANDSCAPE 1011 (NCT04585815) (94)	Phase 1b/2 Open Label Umbrella	Sasanlimab (PD-1 antagonist monoclonal antibody)	Sub-Study A Phase Ib & 2: BRAF V600	Advances NSCLC	Active, Not recruiting
NCT05065398 (95)	Phase 2, Open label, multicenter	HLX208	BRAF V600	Pretreated advanced BRAF NSCLC	Recruiting
NCT03284502 (96)	Phase 1, multicenter	HM95573 with Cobimetinib or Cetuximab	RAF mutant solid tumors Expansion cohort – class II and II BRAF	Advanced RAF positive NSCLC	Recruiting
NCT02974725 (88)	Phase 1b, open label, multicenter	LXH254 with LTT462 or Trametinib or Ribociclib	All BRAF	Advanced or metastatic BRAF or KRAS mutant NSCLC	Active, not recruiting
ENHANCE (97) (NCT05275374)	Phase 1/2a	XP-102; XP-102 with Trametinib	BRAF V600	Advanced BRAF malignant tumors – melanoma, colorectal, NSCLC, thyroid.	Not yet recruiting
B-FAST (82) (NCT03178552)	Phase 2/3, Open label, muticenter, multicohort	Cohort E: Atezolizumab, Cobimetinib, Vemurafenib	Cohort E: BRAF V600	Unresectable, advanced or metastatic BRAF V600 mutation	Recruiting. (Enrollment for cohort E is complete)
NCT04892017 (98)	Phase 1/2, Open label, multicenter	DCC-3116 (monotherapy and in combination with trametinib, binimetinib, or sotorasib	All BRAF	Advanced or metastatic solid tumors with RAS/MAPK pathway mutation	Recruiting
NCT04913285 (99)	Phase 1/2, Open label, multicenter	KIN-2787	All BRAF		Recruiting
NCT03049618 (100)	Phase 2a	sEphB4-HAS (fusion protein) with Pembrolizumab (anti PD-1)	All BRAF	Locally advanced or metastatic non-small cell lung cancer progressed after at least 1 line of platinum-based chemotherapy	Active, Not recruiting
NCT04566393 (101)	Expanded access	Ulixertinib (BVD-523) (ERK1/2 inhibitor)	All BRAF	Advanced NSCLC in altered MAPK pathway	Available
NCT04439279 (102) (MATCH-Subprotocol R)	Phase 2	Trametinib (MEK1/2 inhibitor)	BRAF fusion, non-V600 mutations	Patients With BRAF Fusions, or NonV600E or Non-V600K BRAF Mutations	Active, Not recruiting
NCT04249843 (103)	Phase 1a/1b	BGB 3245 (RAF Dimer inhibitor)	Class II and III BRAF mutations	Advanced or refractory tumore	Recruiting
NCT04488003 (104)	Phase 2, multicenter	Ulixertinib (BVD-523)	BRAF Non-V600	Advanced Malignancies Harboring MEK or Atypical BRAF Alterations	Active, Not recruiting
NCT02428712 (105)	Phase 1/2a	FORE8394	BRAF V600 or Non V600	Advanced unresectable solid tumors	Active, Not recruiting
NCT03843775 (106)	Phase 1/2	Binimetinib and Encorafenib	Non V600 BRAF	Metastatic or advanced-malignant tumors.	Active, Not recruiting

## Conclusion

Precision medicine has revolutionized modern oncology. However, despite the significant progress made in the landscape of NSCLC, treatment for BRAF mutated NSCLC is not satisfactory due to low incidence of this disease. Dabrafenib and Trametinib is the only approved treatment of choice for BRAF-V600E mutated NSCLC and exhibits poor efficacy against non-V600E mutations. As mentioned above, the common mechanisms of resistance for V600E mutant NSCLC involves MAPK reactivation, loss of length BRAF V600E in concert with expression of a truncated form of mutant protein and enhanced EGFR signaling (49). Mechanisms of resistance for BRAF V600E have not been clearly defined. Molecular profiling with next generation sequencing, genomics and single cell sequencing may help in identifying resistance pathways and mutations. Further research is warranted to elucidate and identify mechanisms of resistance in BRAF non-V600E NSCLC and to develop drugs to overcome resistance in BRAF mutations. It is imperative to conduct more clinical trials in future to explore sequencing of therapy and to develop targets targeting resistance of BRAF inhibitors. In future, it will be exciting to see if BRAF inhibitors will have a role in neoadjuvant or adjuvant setting in this group of patients.

## Author contributions

MP wrote the draft of the manuscript. KG and RD reviewed and edited the manuscript. All authors contributed to the manuscript revision, read and approved the submitted version. All authors contributed to the article and approved the submitted version.
